# The difference between dacomitinib and afatinib in effectiveness and safety in first-line treatment of patients with advanced *EGFR*-mutant non-small cell lung cancer: a real-world observational study

**DOI:** 10.1186/s12885-024-11956-w

**Published:** 2024-02-19

**Authors:** Wen-Chien Cheng, Chi-Chien Lin, Wei-Chih Liao, Yu-Chao Lin, Chia-Hung Chen, Hung-Jen Chen, Chih-Yen Tu, Te-Chun Hsia

**Affiliations:** 1 Division of Pulmonary and Critical Care, Department of Internal Medicine, China Medical University Hospital, Taichung, Taiwan; 2 School of Medicine, College of Medicine, China Medical University, Taichung, Taiwan; 3 Department of Life Science, National Chung Hsing University, Taichung, Taiwan; 4 PhD Program in Translational Medicine, National Chung Hsing University, Taichung, Taiwan; 5 Rong Hsing Research Center for Translational Medicine, National Chung Hsing University, Taichung, Taiwan; 6 Institute of Biomedical Science, the iEGG and Animal Biotechnology Center, Advanced Plant and Food Crop Biotechnology Center, National Chung-Hsing University, Taichung, Taiwan; 7 Department of Pharmacology, College of Medicine, Kaohsiung Medical University, Kaohsiung, Taiwan; 8 Department of Medical Research, Taichung Veterans General Hospital, Taichung, Taiwan

**Keywords:** Non-small cell lung cancer (NSCLC), Epidermal growth factor receptor (EGFR), Dacomitinib, Afatinib

## Abstract

**Objectives:**

The irreversible epidermal growth factor receptor tyrosine kinase inhibitors (EGFR TKIs) afatinib and dacomitinib are approved for first-line treatment of *EGFR* mutation-positive non-small cell lung cancer (NSCLC). We aimed to compare the efficacy and safety of afatinib and dacomitinib in this setting.

**Materials and methods:**

Between September 2020 and March 2023, we retrospectively recruited patients diagnosed with advanced-stage *EGFR*-mutant NSCLC who were treated with first-line irreversible EGFR-TKIs. The enrolled patients were assigned to two groups based on whether they received afatinib or dacomitinib.

**Results:**

A total of 101 patients were enrolled in the study (70 to afatinib and 31 to dacomitinib). The partial response rates (PR) for first-line treatment with afatinib and dacomitinib were 85.7 and 80.6% (*p* = 0.522). The median progression-free survival (PFS) (18.9 vs. 16.3 months, *p* = 0.975) and time to treatment failure (TTF) (22.7 vs. 15.9 months, *p* = 0.324) in patients with afatinib and dacomitinib treatment were similar. There was no significant difference observed in the median PFS (16.1 vs. 18.9 months, *p* = 0.361) and TTF (32.5 vs. 19.6 months, *p* = 0.182) between patients receiving the standard dose and those receiving the reduced dose. In terms of side effects, the incidence of diarrhea was higher in the afatinib group (75.8% vs. 35.5%, *p* <  0.001), while the incidence of paronychia was higher in the dacomitinib group (58.1% vs. 31.4%, *p* = 0.004). The PFS (17.6 vs. 24.9 months, *p* = 0.663) and TTF (21.3 vs. 25.1 months, *p* = 0.152) were similar between patients younger than 75 years and those older than 75 years.

**Conclusion:**

This study showed that afatinib and dacomitinib had similar effectiveness and safety profiles. However, they have slightly different side effects. Afatinib and dacomitinib can be safely administered to patients across different age groups with appropriate dose reductions.

## Introduction

Lung cancer continues to be one of the most widespread and fatal cancers globally [1]. Treatment strategies for advanced non-small cell lung cancer (NSCLC) are now personalized and guided by molecular tests. Studies show that patients with specific mutations in lung adenocarcinoma who receive matched targeted therapies experience longer overall survival (OS) [2]. *EGFR* mutations, found in 30 to 50% of lung adenocarcinomas, commonly include exon 19 deletions (in 45% of patients) and the exon 21 L858R mutation (in 40% of patients). These are known as sensitizing *EGFR* mutations [3]. Epidermal growth factor receptor-tyrosine kinase inhibitors (EGFR-TKIs) have greatly improved the prognosis and quality of life for NSCLC patients with *EGFR* mutations, making them the first-line standard treatment over cytotoxic chemotherapy [4]. Three generations of EGFR-TKIs are available. First-generation EGFR-TKIs (erlotinib and gefitinib) reversibly block ATP-binding sites, stopping downstream signaling. Second-generation EGFR-TKIs (afatinib and dacominitib) form irreversible bonds with ErbB receptors, inhibiting signaling and offering an alternative for acquired resistance to first-generation TKIs. Third-generation EGFR-TKIs (osimertinib) treat T790M *EGFR*-mutant tumors, which represent the most common resistance mechanism, occurring in approximately 50% of patients who have used first- and second-generation EGFR-TKIs [5].

The selection of these three generations of drugs as first-line treatment is an important issue. In the case of first-generation EGFR-TKIs, several studies demonstrated that gefitinib and erlotinib had comparable efficacy, with gefitinib exhibiting a more favorable safety profile than erlotinib [6–8]. In the LUX-Lung 7 study, the irreversible ErbB family blocker afatinib notably improved results in *EGFR*-mutated NSCLC treatment-naive patients compared to gefitinib [9]. Dacomitinib, another irreversible ErbB family blocker, significantly enhanced progression-free survival (PFS) compared to gefitinib in the first-line treatment of *EGFR* mutation-positive NSCLC patients [10]. In the FLAURA study, first-line osimertinib treatment provided a clinically significant improvement in both PFS and OS compared to first-generation EGFR TKIs [11, 12]. The new generation of EGFR-TKIs appears to offer better clinical efficacy than first-generation EGFR-TKIs. However, there have been no randomized controlled trials (RCTs) comparing second- and third-generation EGFR-TKIs. A few real-world studies do not strongly favor osimertinib over afatinib in terms of longer median PFS and OS in first-line treatment [13, 14]. Osimertinib was effective in patients with brain metastasis, while afatinib demonstrated potential benefits in patients with the L858R mutation who did not have brain metastasis [14]. Since subsequent treatment after the failure of third-generation EGFR-TKIs is not well established, sequential afatinib and osimertinib showed promise in Asian NSCLC patients with *EGFR* mutations and T790M-mediated resistance, especially in those with Del19-positive disease [15]. Therefore, using second-generation EGFR-TKIs as a first-line follow-up to third-generation EGFR-TKIs remains a favorable treatment option. In addition to assessing medication efficacy and determining subsequent treatment strategies, factors such as patients’ tolerance to medication side effects, for example, the higher toxicity of second-generation EGFR-TKIs, and regulations within each country’s healthcare system regarding medications, for instance, the limited coverage of third-generation EGFR-TKIs, as well as physicians’ medication preferences can influence first-line treatment decisions.

There is currently limited research comparing these 2 second-generation EGFR TKIs. Li et al. [16] reported that in patients with NSCLC carrying uncommon *EGFR* mutations, dacomitinib displayed more favorable activity with manageable toxicity and distinct progression patterns compared to afatinib. To the best of our knowledge, there are currently no studies comparing the clinical treatment outcomes of these 2 second-generation EGFR-TKIs in NSCLC patients with common EGFR mutations. The purpose of this study was to compare the therapeutic effectiveness and adverse effects of afatinib and dacomitinib in NSCLC patients with common *EGFR* mutations.

## Material and methods

### Eligible patients

The retrospective observational study was conducted at China Medical University Hospital, a leading tertiary referral center in Taiwan, spanning the period from September 2020 to March 2023. We exclusively considered patients classified as stage IIIB-IV NSCLC based on the American Joint Committee on Cancer, 8th edition, harboring an *EGFR* exon 19 deletion or exon 21 L858R point mutation. Patients who underwent first-line treatment with second-generation EGFR-TKIs (afatinib or dacomitinib) were included in the study. Patients who fell into the following categories were excluded: patients who did not undergo 2nd generation EGFR-TKI treatment, patients with uncommon EGFR mutations, and patients for whom data were insufficient for analysis. Baseline data for each patient, including age, sex, smoking history, Eastern Cooperative Oncology Group Performance Status (ECOG PS), TNM stage at initial diagnosis, distant metastasis patterns, *EGFR* mutation subtype, treatment-related adverse effects, and treatment duration, were extracted from electronic medical records. The study was approved by the institutional ethics committee (IRB number: CMUH110-REC1–244), and informed consent was waived because of the study’s retrospective nature. The study adhered to the Declaration of Helsinki, and individual data were anonymized prior to inclusion in this research. Additionally, the study did not receive funding from any commercial entity.

### Effectiveness assessment

In our study, all patients underwent a comprehensive imaging assessment, which included regular computed tomography (CT) examinations, brain imaging via CT or magnetic resonance imaging (MRI) in response to any neurological symptom changes, and positron emission tomography (PET) scans for initial staging. Furthermore, after EGFR-TKI therapy was initiated, chest CT scans were scheduled at 3-month intervals to monitor tumor response. The study employed the Response Evaluation Criteria in Solid Tumors (RECIST) criteria [17] to evaluate treatment response. Patients were monitored for a three-month period, during which their target lesion size changes were assessed through imaging studies. Depending on these evaluations, the disease status was categorized into complete response (CR), partial response (PR), stable disease (SD), or progressive disease (PD). We compared and calculated the progression-free survival (PFS) and time to treatment failure (TTF) for both drugs. PFS refers to the time from initiation of EGFR-TKI treatment to the occurrence of disease progression or death. TTF represents the period starting from the initiation of first-line EGFR-TKI treatment and ending at the discontinuation of first-line EGFR-TKI. Adverse drug reactions (ADRs) were detected via electronic medical records, and their severity was evaluated using the Naranjo ADR Probability Scale [18].

### EGFR mutation analysis

Tumor tissue samples were obtained from NSCLC patients during initial diagnosis or upon rebiopsy following disease progression, following standard clinical protocols. Formalin-fixed, paraffin-embedded (FFPE) tissue blocks were sectioned into 5-μm-thick slices and stored in sterile Eppendorf tubes, containing 10–100% cancer cells. DNA extraction from the FFPE tumor tissue sections was performed using the spin column-based cobas DNA sample preparation kit (Roche Molecular Systems, Inc., South Branchburg, USA), as per the manufacturer’s instructions. DNA eluates’ concentration and purity were assessed using spectroscopy and fluorometry, following manufacturers’ protocols and laboratory guidelines [19]. The EGFR mutation status in tumor tissue was determined using the cobas® EGFR Mutation Test v2 kit (Roche).

Liquid biopsy was utilized to detect the T790M mutation in cases where patients were unable to undergo tissue rebiopsy. Ten milliliters of blood were collected into circulating cell-free DNA (CfDNA) collection tubes and centrifuged at 3000 rpm for 20 minutes at room temperature within 36 hours of collection. Plasma samples were processed, and CfDNA was isolated using the cobas® CfDNA sample preparation kit. The target DNA was then amplified and detected on cobas z 480 analyzers using the amplification and detection reagents provided in the cobas® EGFR Mutation Test v2 kit (Roche) [20].

### Statistical analyses

All data were subjected to statistical analyses using MedCalc for Windows version 18.10 (MedCalc Software, Ostend, Belgium). Continuous variables are presented as either the mean ± standard deviation (SD) or the median and interquartile range (IQR) for normally and nonnormally distributed data, respectively. Group differences were assessed using the t test for normally distributed continuous data and the Kruskal–Wallis test for nonnormally distributed and ordinal data. Categorical variables were expressed as counts and percentages and analyzed with the Chi-square test or Fisher’s exact test. Survival analyses, including progression-free survival (PFS) and TTF, were conducted using the Kaplan–Meier method. The results are presented as HRs with 95% confidence intervals (CIs). Significance was set at a threshold of *p* <  0.05.

## Results

### Baseline characteristics of patients receiving afatinib and dacomitinib as first-line treatment

**Fig. 1:**
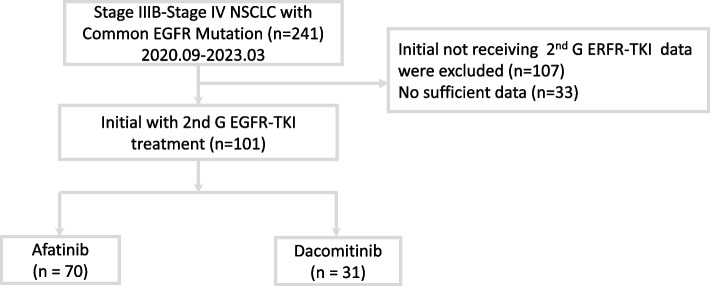
Flowchart for patient enrollment EGFR, epidermal growth factor receptor; TKI, tyrosine kinase inhibitor; NSCLC, non-small cell lung cancer; 2nd G, second generation

From September 2020 to March 2023, 241 patients were diagnosed with stage IIIB-IV NSCLC with *EGFR* mutations. Among them, 101 patients who received 2nd generation EGFR-TKIs (70 with afatinib, 31 with dacomitinib) as their first-line treatment were enrolled in the final analysis (Fig. 1). Among the 101 patients, 41 (40.6%) were male, and 38 (37.6%) were smokers. No significant differences were observed in age, sex, smoking status, ECOG PS, the pattern of distant metastasis or response to the initial treatment between the two groups. In the current study, only 29 patients (28.7%) received the standard dose of irreversible EGFR-TKIs as their first-line therapy (afatinib 40 mg or dacomitinib 45 mg). Afatinib was used more often at a standard dose, and dacomitinib was used more often at a lower dose, but the difference was not significant (34.3% vs. 16.1%; *p* = 0.064). Treatment discontinuation due to side effects showed no significant difference between the groups (12.9% vs. 4.3%; *p* = 0.118). In the entire cohort, 29 patients (28.7%) underwent radiotherapy for local control. Among them, 12 out of 31 patients (38.7%) in the dacomitinib group received radiotherapy, while 17 out of 70 patients (24.3%) in the afatinib group underwent radiotherapy. (Table 1).

**Table 1:** Patient Characteristics

	All (*n* = 101)	Dacomitinib (*n* = 31)	Afatinib(*n* = 70)	*p*-value
**Age ≥ 65 years**	53 (52.5)	19 (61.3)	34 (48.6)	0.240
**Male**	41 (40.6)	10 (32.3)	31 (44.3)	0.257
**Smoking**	38 (37.6)	10 (32.3)	28 (40.0)	0.461
**ECOG PS** **≥** **2**	3 (3.0)	1 (3.2)	2 (2.9)	1.000
***EGFR*** **mutation**				0.293
**Del 19**	37 (36.6)	9 (29.0)	28 (40.0)	
**L858R**	64 (63.4)	22 (71.0)	42 (60.0)	
**Metastasis organ**				
**Lung metastasis**	34 (33.7)	7 (22.6)	27 (38.6)	0.119
**LN metastasis**	79 (78.2)	24 (77.4)	55 (78.6)	0.897
**Pleural metastasis**	46 (45.5)	16 (51.6)	30 (42.9)	0.417
**Liver metastasis**	12 (11.9)	5 (16.1)	7 (10.0)	0.382
**Bone Metastasis**	39 (38.6)	13 (41.9)	26 (37.1)	0.649
**CNS metastasis**	13 (12.9)	1 (3.2)	12 (17.1)	0.061
**Adrenal metastasis**	5 (5.0)	2 (6.5)	3 (4.3)	0.641
**EGFR-TKI Treatment**				
**Standard dose**	29 (28.7)	5 (16.1)	24 (34.3)	0.064
**Adjust dose**	12 (11.9)	4 (12.9)	8 (11.4)	1.000
**Discontinuation**	7 (6.9)	4 (12.9)	3 (4.3)	0.118
**Local radiation therapy**	29 (28.7)	12 (38.7)	17 (24.3)	0.147
**Response**				0.522
**Partial response**	85 (84.2)	25 (80.6)	60 (85.7)	
**Stable disease**	16 (15.8)	6 (19.4)	10 (14.3)	

*CNS* central nervous system: *ECOG PS* Eastern Cooperative Oncology Group performance status: *EGFR* epidermal growth factor receptor: *LN* Lymph Node: *TKI* tyrosine kinase inhibitor

Continuous variables are presented as the mean (standard deviation) or median (interquartile range); categorical variables are presented as the number and percentage

### Effectiveness of afatinib and dacomitinib as first-line treatments

**Fig. 2:**
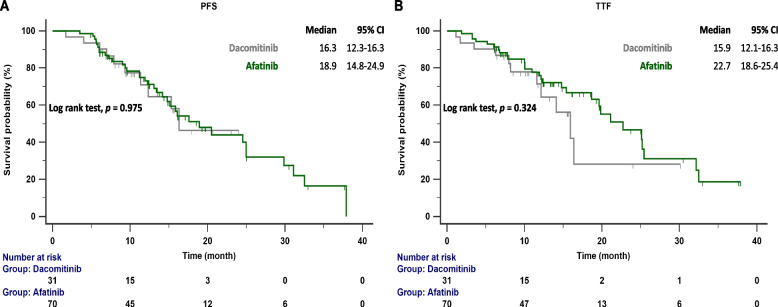
**A** PFS in patients with *EGFR*-mutant NSCLC treated with dacomitinib and afatinib; **B** TTF in patients with *EGFR*-mutant NSCLC treated with dacomitinib and afatinib. EGFR, epidermal growth factor receptor; PFS, progression-free survival; TTF, time to treatment failure

**Fig. 3:**
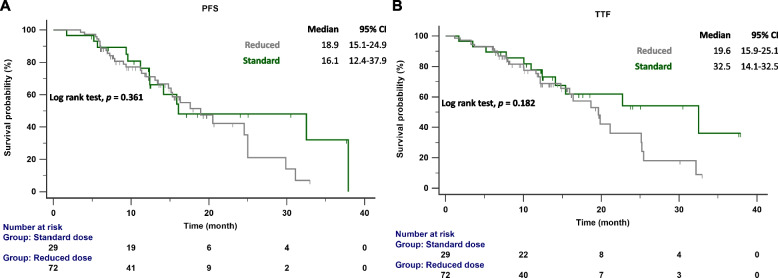
**A** PFS in patients with *EGFR*-mutant NSCLC treated with standard dose and reduced dose of 2nd G EGFR-TKIs; **B** TTF in patients with *EGFR*-mutant NSCLC treated with standard dose and reduced dose of 2nd G EGFR-TKIs. EGFR, epidermal growth factor receptor; PFS, progression-free survival; TTF, time to treatment failure; TKI, tyrosine kinase inhibitor

**Fig. 4:**
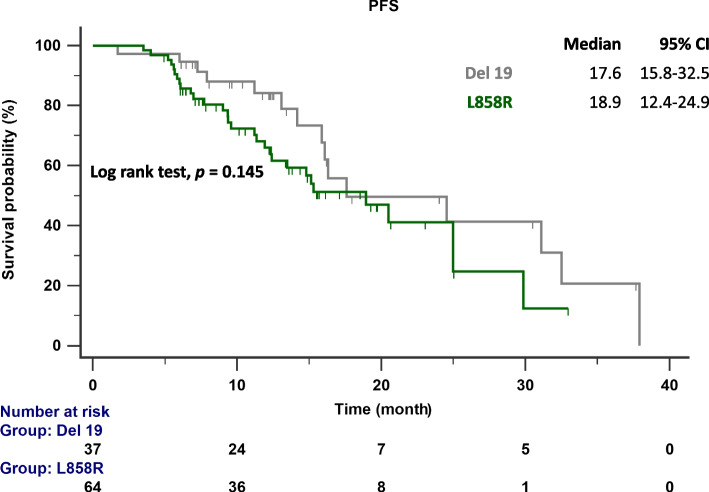
PFS in patients with different *EGFR*-mutant NSCLC subtypes (del19 vs. L858R) treated with 2nd G EGFR-TKIs EGFR, epidermal growth factor receptor; PFS, progression-free survival; TKI, tyrosine kinase inhibitor

**Fig. 5:**
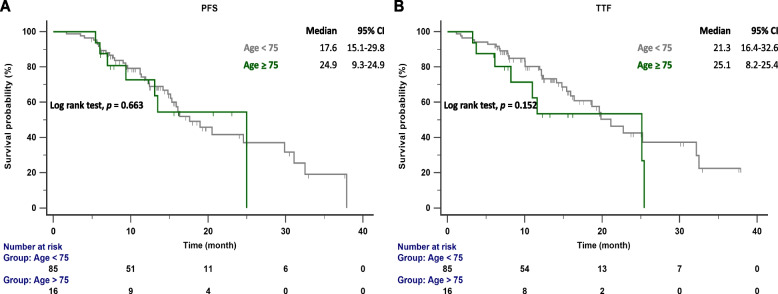
**A** PFS in *EGFR*-mutant NSCLC patients in different age groups (age ⩾75 years vs. age < 75 years) treated with 2nd G EGFR-TKIs; **B** TTFs in *EGFR*-mutant NSCLC patients in different age groups (age ⩾75 years vs. age < 75 years) treated with 2nd G EGFR-TKIs. EGFR, epidermal growth factor receptor; PFS, progression-free survival; TTF, time to treatment failure

The follow-up ended on October 1, 2023. The median follow-up time was 15.7 months (range 13.6–19.2 months). According to RECIST criteria, we compared the initial responses to first ling treatment with afatinib and dacomitinib, and no significant difference was observed (85.7% vs. 80.6%) (Table 1). The PFS and TTF for each drug were determined based on the number of months without disease progression and discontinuation of EGFR TKI treatment, and these were compared (Fig. 2A and B). No significant difference was observed between the median PFS (16.3 months vs. 18.9 months; *p* = 0.975) and TTF (15.9 months vs. 22.7 months; *p* = 0.324) of dacomitinib and afatinib, indicating similar effectiveness in NSCLC patients. We also compared the effectiveness between reduced and standard doses of afatinib or dacomitinib. The PFS (16.1 months vs. 18.9 months; *p* = 0.361) and TTF (32.5 months vs. 19.6 months; *p* = 0.182) between standard and reduced doses showed no significant difference (Fig. 3A and B). No significant difference in PFS was observed between patients with del19 and L858R mutations receiving 2nd generation EGFR-TKI treatment (17.6 months vs. 18.9 months; *p* = 0.145) (Fig. 4). In patients aged < 75 years versus ⩾75 years, the median PFS was 17.6 months versus 24.9 months (*p* = 0.663), and the median TTF was 21.3 months versus 25.1 months. (*p* = 0.152) (Fig. 5A and B).

### The toxicities of afatinib and dacomitinib

Table 2 illustrates the major toxicities of both drugs. The most frequently observed adverse reactions during EGFR-TKI (afatinib and dacomitinib) therapy included skin rash/acneiform eruption, stomatitis/oral ulcer, diarrhea, paronychia, and pruritus. Diarrhea was more prevalent in the afatinib group (75.8% vs. 35.5%, *p* < 0.001), whereas paronychia was more common in the dacomitinib group (58.1% vs. 31.4%, *p* = 0.004). There were no significant differences in other side effects between the two drugs.

**Table 2:** Adverse Event of dacomitinib and afatinib

		Dacomitinib (n = 31)	Afatinib (n = 70)	*p*-value
**Skin Rash/ Acne**				0.376
** Grade1–2**	83 (82.2)	25 (80.6)	58 (82.9)	
** Grade > 2**	3 (3.0)	0 (0)	3 (4.3)	
**Stomatitis/oral ulcer**				0.547
** Grade1–2**	42 (41.6)	15 (48.4)	27 (38.6)	
** Grade > 2**	1 (1.0)	0 (0)	1 (1.4)	
**Diarrhea**				< 0.001
** Grade1–2**	62 (61.4)	11 (35.5)	51 (72.9)	
** Grade > 2**	2(2.0)	0(0)	2(2.9)	
**Paronychia**				0.004
** Grade 1–2**	27 (26.7)	15 (48.4)	12 (17.1)	
** Grade > 2**	13 (14.3)	3 (9.7)	10 (14.3)	
**Pruritus**				0.141
** Grade 1–2**	72 (71.3)	19 (61.3)	53 (75.7)	
** Grade > 2**	0 (0)	0 (0)	0 (0)	

### Treatment pattern after disease progression

**Fig. 6:**
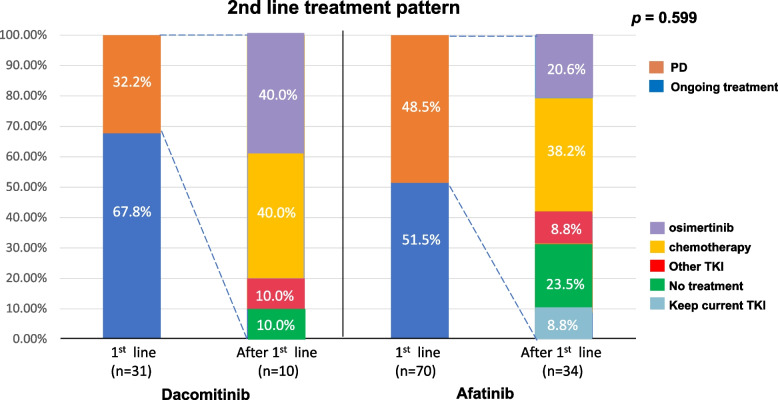
Subsequent treatment regimen for patients who experienced disease progression while on dacomitinib or afatinib

Out of 44 (43.6%) patients who experienced disease progression following first-line EGFR-TKI treatment, 34 (47.8%) were treated with afatinib and 10 (32.5%) with dacomitinib until the end date of the follow-up period (October 1, 2023). Out of 44 patients, 32 (72.7%) underwent rebiopsy due to disease progression. In the dacomitinib group, 8 out of 10 patients (80%) underwent rebiopsy (4 with tissue and liquid biopsy; 4 with tissue biopsy only), revealing 3 patients (37.5%) with T790M mutation. In the afatinib group, 24 out of 34 patients (70.6%) underwent rebiopsy (5 with tissue and liquid biopsy; 19 with tissue biopsy only), with 4 patients (16.7%) showing T790M mutation. There was no significant difference in T790M mutation occurrence between patients treated with dacomitinib (37.5%) and afatinib (16.7%) after disease progression (*p* = 0.224). The subsequent treatment pattern after progression of dacomitinib and afatinib was not significantly different (*p* = 0.599). It is worth noting that the subsequent switch to osimertinib was seen in 40% of patients on dacomitinib, while it was observed in 20.6% of patients on afatinib. Although the number of afatinib cases was relatively low, it did not reach statistical significance. In the afatinib group, there were more patients without subsequent treatment, which could be due to the potential impact of not performing rebiopsies on the occurrence of T790M mutations (Fig. 6).

## Discussion

This study is the first to explore the clinical effectiveness of dacomitinib and afatinib in Taiwanese NSCLC patients with common *EGFR* mutations. Our findings reveal that both afatinib and dacomitinib treatment achieved comparable median PFS and TTF in real-world practice. Afatinib and dacomitinib have slightly different drug side effects. Furthermore, no disparity in median PFS and TTF was observed between the standard and reduced dose groups. Among patients, there was no significant difference in median PFS and TTF when comparing elderly (age ⩾75 years) and younger individuals (age < 75 years) receiving 2nd generation EGFR-TKIs as first-line treatment.

Dacomitinib and afatinib, both second-generation EGFR-TKIs, irreversibly disrupt the signaling of the pan-Erb B family of receptors [21, 22]. In vitro studies show that afatinib and dacomitinib have lower 50% inhibitory concentrations against *EGFR*-mutant cell lines compared to first-generation EGFR-TKIs such as gefitinib and erlotinib [23, 24]. Existing evidence supports that in individuals with common *EGFR* mutations and NSCLC, 2nd generation TKIs (afatinib or dacomitinib) are associated with improved PFS compared to the 1st generation TKI gefitinib [9, 10]. Another pooled subset analysis from two randomized trials has shown promising trends, indicating that dacomitinib may offer superior PFS compared to erlotinib, particularly for patients with EGFR activation mutations [25]. Afatinib outperformed erlotinib as a second-line treatment for advanced squamous cell carcinoma, showing improved PFS and OS [26]. Huang et al. [27] confirmed that in real-world practice, afatinib reduced the risk of progression compared to first-generation EGFR-TKIs, with an HR of 0.73 (95% CI 0.57–0.94; *p* = 0.017). According to the aforementioned studies, it appears that both afatinib and dacomitinib, both second-generation EGFR-TKIs, exhibit similar efficacy. However, there is a lack of both clinical trials and real-world studies directly comparing the 2 second-generation EGFR-TKIs.

For further clarification of the disparities in clinical efficacy between dacomitinib and afatinib, Li et al. [16] demonstrated that dacomitinib exhibited a more favorable response with manageable side effects and distinct progression patterns in individuals with NSCLC bearing uncommon *EGFR* mutations. In the current study, we found that afatinib and dacomitinib offer comparable PFS and TTF in NSCLC patients with common *EGFR* mutations. We did not include uncommon *EGFR* mutations in our analysis, so the results differ slightly. Dacomitinib and afatinib exhibited a similar range of adverse events, primarily encompassing rash, diarrhea, oral mucositis, paronychia, and dry skin with itching. In the current study, we observed some differences in side effects between dacomitinib and afatinib. Dacomitinib was associated with a higher incidence of paronychia (58.1% vs. 31.4%; *p* = 0.004), whereas afatinib was associated with a greater likelihood of experiencing diarrhea (75.8% vs. 35.5%; *p* < 0.001). Li et al. [16] also found higher rates of grade 1 adverse events with dacomitinib than with afatinib (*p* = 0.006). However, grade 3 diarrhea occurred significantly more frequently with afatinib than with dacomitinib (*p* = 0.036). This may be related to the initial standard dose of treatment. In this study, dacomitinib was used at a standard dose less frequently than afatinib. However, the study by Li et al. [16] did not provide information on initial dose and dose adjustments.

Dose reductions were necessary for 53.3% of patients on 40 mg afatinib daily in the LUX-Lung 3 trial and 28.0% in the LUX-Lung 6 trial. Lowering the dose to 30 mg daily reduced ADR incidence while maintaining comparable PFS in subanalyses of these trials [28]. In the ARCHER 1050 study, dacomitinib dose reduction was necessary in 66% of the patients due to intolerable adverse events [10]. Dose reductions of dacomitinib also helped manage adverse events, and the PFS and OS benefits remained for patients with dose reductions of dacomitinib [29]. A meta-analysis also showed that the 30 mg afatinib dose led to fewer severe adverse reactions in NSCLC patients, with comparable effectiveness for those without brain metastasis [30]. Li et al. [31] demonstrated that 65.6% of patients who received an initial treatment dose of 30 mg dacomitinib showed favorable responses in NSCLC patients with uncommon *EGFR* mutations. In this study, only 29 patients (28.7%) received the initial standard dose of irreversible EGFR-TKIs as their first-line therapy. When comparing the standard doses of the second-generation EGFR-TKIs (40 mg of afatinib or 45 mg of dacomitinib) to the reduced-dose groups, we found that PFS and TTF were similar. This result demonstrates that using reduced doses of second-generation EGFR-TKIs to minimize drug-related side effects helps patients maintain their treatment without interruptions, resulting in better treatment outcomes.

The use of second-generation EGFR-TKIs in older patients is also a noteworthy consideration. In a subgroup analysis of the LUX-Lung 3, 6, and 7 trials, afatinib proved effective and well tolerated in *EGFR* mutation-positive NSCLC patients, irrespective of their age at diagnosis [32]. In the NEJ027 study, dose adjustments (78.9%) were common in older Japanese patients with *EGFR* mutation-positive NSCLC, but treatment discontinuation (21.1%) was rare, allowing most to continue treatment for over a year [33]. Chang et al. [34] conducted a study to assess the effectiveness of EGFR-TKIs in older patients, including those with a poor Eastern Cooperative Oncology Group (ECOG) performance status (PS), and showed that afatinib as a first-line treatment was associated with a longer PFS. This study also found that using the second-generation EGFR-TKI, the obtained PFS and TTF were comparable in both age groups, those aged 75 and older and those younger than 75. This suggests that second-generation EGFR-TKIs are effective and safe in older patients.

Patients with common *EGFR* mutations (such as exon 19 deletions and exon 21 L858R mutations) significantly benefit from EGFR-TKIs; common *EGFR* mutations constitute over 85% of cases, while uncommon *EGFR* mutations (within exons 18–21) make up the remaining 10–15% [35]. Recent developments in NGS show that approximately 10% of patients have compound *EGFR* mutations, meaning multiple distinct *EGFR* genetic changes initially [36]. Kohsaka et al. [37] reported that *EGFR* compound mutations were detected in 15.9% of 390 *EGFR*-mutated NSCLC specimens. Notably, L858R exhibited a higher rate (19.5%) than Del19 (4.7%).Patients with compound *EGFR* mutations tend to be less responsive to TKI therapies than those with a single *EGFR* mutation [38, 39]. Yang et al.’s [40] studies confirmed afatinib’s efficacy in NSCLC with major uncommon mutations (G719X, S768I, L861Q). Li et al. [16, 31] reported dacomitinib’s efficacy in NSCLC patients with uncommon *EGFR* mutations, both in first-line and later-line treatments. Our study found that patients with L858R and Del19 mutations had comparable PFS when receiving second-generation EGFR-TKIs as first-line treatment. This suggests that L858R patients, who may have compound mutations, might benefit from second-generation EGFR-TKIs for improved treatment outcomes.

Although our study is the first study in Taiwan comparing afatinib and dacomitinib in NSCLC patients with common *EGFR* mutations, it does have several limitations. First, this study is limited by being single center-based in Taiwan and by its retrospective design, potentially introducing more bias compared to prospective studies. Therefore, our results might not be applicable to different ethnic groups. Second, due to the relatively small sample size in our study, we need to interpret the data from subgroup analyses with caution. Third, the clinical physicians determined the initial administration and dosage of second-generation EGFR-TKIs, representing another potential bias in our study. Consequently, only 28.7% of the patients received the standard dosage of EGFR-TKIs, potentially affecting treatment efficacy and side effects. Finally, the rate of T790M mutation and subsequent treatment with osimertinib in the dacomitinib group was slightly higher than that in the afatinib group, but it did not reach statistical significance. This could be influenced by whether patients underwent rebiopsy and their willingness to receive subsequent treatment. Despite these limitations, our study provides valuable evidence that another second-generation EGFR-TKI, dacomitinib, offers clinical treatment efficacy similar to afatinib, with slight differences in side effects. However, larger or even prospective studies may be needed to verify the similarities and differences between these two drugs.

## Conclusion

The study findings suggest that afatinib and dacomitinib are similarly effective and have comparable safety profiles. However, there are slight differences in their side effects. Notably, both drugs can be administered safely across different age groups, and dose adjustments can be made to extend treatment and survival.

## Data Availability

Upon reasonable request, the corresponding author is willing to provide the datasets used and/or analyzed in the current study.

## Abbreviations

CT: computed tomography
EGFR: epidermal growth factor receptor
EGFR-TKI: epidermal growth factor receptor tyrosine kinase inhibitor
ECOG: Eastern Cooperative Oncology Group
MRI: magnetic resonance imaging
NSCLC: non-small cell lung cancer
ORR: objective response rate
OS: overall survival
PET: positron emission tomography
PFS: progression-free survival
PS: performance status
TKI: tyrosine kinase inhibitor
TTF: time to treatment failure
